# Investigating the Effect of Reflectance Tuning on Photocatalytic Dye Degradation with Biotemplated ZnO Photonic Nanoarchitectures Based on *Morpho* Butterfly Wings

**DOI:** 10.3390/ma16093584

**Published:** 2023-05-07

**Authors:** Gábor Piszter, Gergely Nagy, Krisztián Kertész, Zsófia Baji, Krisztina Kovács, Zsolt Bálint, Zsolt Endre Horváth, József Sándor Pap, László Péter Biró

**Affiliations:** 1 Institute of Technical Physics and Materials Science, Centre for Energy Research, 29-33 Konkoly Thege Miklos St., 1121 Budapest, Hungaryhorvath.zsolt.endre@ek-cer.hu (Z.E.H.);; 2 Surface Chemistry and Catalysis Department, Institute for Energy Security and Environmental Safety, Centre for Energy Research, 29-33 Konkoly Thege Miklos St., 1121 Budapest, Hungary; 3 Radiation Chemistry Department, Institute for Energy Security and Environmental Safety, Centre for Energy Research, 29-33 Konkoly Thege Miklos St., 1121 Budapest, Hungary; 4 Department of Zoology, Hungarian Natural History Museum, 13 Baross St., 1088 Budapest, Hungary

**Keywords:** atomic layer deposition, biotemplating, butterfly wing, photocatalysis, photonic nanoarchitecture, structural color

## Abstract

Photonic nanoarchitectures of butterfly wings can serve as biotemplates to prepare semiconductor thin films of ZnO by atomic layer deposition. The resulting biotemplated ZnO nanoarchitecture preserves the structural and optical properties of the natural system, while it will also have the features of the functional material. The ZnO-coated wings can be used directly in heterogeneous photocatalysis to decompose pollutants dissolved in water upon visible light illumination. We used the photonic nanoarchitectures of different *Morpho* butterflies with different structural colors as biotemplates and examined the dependence of decomposition rates of methyl orange and rhodamine B dyes on the structural color of the biotemplates and the thickness of the ZnO coating. Using methyl orange, we measured a ten-fold increase in photodegradation rate when the 20 nm ZnO-coated wings were compared to similarly coated glass substrates. Using rhodamine B, a saturating relationship was found between the degradation rate and the thickness of the deposited ZnO on butterfly wings. We concluded that the enhancement of the catalytic efficiency can be attributed to the slow light effect due to a spectral overlap between the ZnO-coated *Morpho* butterfly wings reflectance with the absorption band of dyes, thus the photocatalytic performance could be changed by the tuning of the structural color of the butterfly biotemplates. The photodegradation mechanism of the dyes was investigated by liquid chromatography–mass spectroscopy.

## 1. Introduction

Biotemplating made possible the production of artificial materials that utilize a wide range of elements of the periodic table [1,2,3] in the form of intricate structures typical of biological systems [4]. With this technique, novel functional materials can be fabricated that combine the complex hierarchy of biological structures with the advantages of artificial materials [5]. These new inorganic functional materials are produced by incorporating the desired compounds into the templates created by nature, optionally followed by mineralization processes that may remove the templates through degradation [6]. It is especially exciting if photonic-crystal-type nanoarchitectures form the basis of the biological templates [7,8,9]. These structures are widespread in living organisms and often found in the cuticle of insects [10,11,12], producing eye-catching structural colors by the wavelength-selective reflection of incident white light.

Structural colors generated by photonic nanoarchitectures perform important functions in the life of these animals [13,14,15,16]; therefore, they have been perfected over millions of years [17] in the form of diverse nanostructures with extraordinary optical properties [10,11,12]. Photonic nanoarchitectures in butterfly wings are constituted of two non-absorbent materials—chitin and air—with different refractive indices that can form a photonic band gap if their periodicity is comparable with the wavelength of visible light [18]. The optical characteristics of the structural color depend on the structural parameters and the refractive index contrast of the photonic nanoarchitecture. Therefore, by modifying the material properties or ratio of the constituents, the spectral characteristics of reflected light can be changed [19]; both additive and subtractive modification of butterfly wing scale nanostructures result in a color shift of the wings when atomic layer deposition of thin films or oxygen plasma etching is applied, respectively [20]. If a suitable semiconductor thin film coating is deposited onto the photonic nanoarchitecture in butterfly wing scales, they can be used even in heterogeneous photocatalysis for the controlled decomposition of substances dissolved in water by sunlight [9,19,21,22].

In a semiconductor-based photocatalytic process, photogenerated electrons and holes drive the reduction and oxidation, respectively, of compounds adsorbed on the surface of a photocatalyst [23]. The efficiency of converting solar to chemical energy can be greatly enhanced by increasing the effective surface area of the photocatalyst, or by improving the efficiency of the photoexcitation process [24,25,26,27]. These two goals can be achieved simultaneously with the biotemplated semiconductors made of structurally colored butterfly wings. The number of adsorbed molecules significantly increases with the micro- or nanostructured surface [7,8,9], and at the same time, the semiconductor is able to interact more efficiently with the incident photons due to the slow light effect by tuning the photonic band gap to the absorption band of the dye to be decomposed [28]. It was reported recently, that TiO_2_-coated nanoporous alumina Bragg reflectors had maximal photocatalytic enhancement when the red edge of the photonic band gap was spectrally close to the red or blue edge of the absorption band of the dye [29]. On the other hand, the central part of the dye absorption band itself greatly reduced the intensity of the light falling on the photonic nanoarchitecture due to a screening effect.

It was also shown that slow photons at the blue edge of the photonic band gap are able to enhance light harvesting through the loose confinement of the field, which resulted in enhanced absorption [30]. These results together with our recent findings that the overlap between the reflectance peak of the photonic nanoarchitecture and the dye absorption determines the efficiency of dye decomposition on biotemplated ZnO photonic nanoarchitectures [12] clearly show the importance of the proper spectral tuning of the biotemplated semiconductor thin films in photocatalytic applications.

In the present work, we utilized photonic nanoarchitectures of *Morpho* butterflies as biotemplates. According to our previous studies, the *Morpho*-based templates were the most effective photocatalysts [12]. Therefore, we extended our investigations to four butterfly species representing this genus, with similar photonic nanoarchitectures but with different structural colors (from deep blue to greenish blue). To our best knowledge, it is for the first time that biotemplates with similar nanostructures, but with different optical properties are used within the same experimental setup, to test the relation of the different optical properties with the photocatalytic performance. We examined how the decomposition rate of methyl orange (MO), and rhodamine B (RhB) depends on the structural color of the biotemplates and the thickness of the applied ZnO coating upon visible light illumination. We found that the overlap of the blue and red edges of the photonic band gap with the absorption band of the dyes plays an important role in the enhancement of the photocatalytic activity. Our findings may facilitate the application of biotemplated semiconductor photonic nanoarchitectures as a bio-inspired testing platform of photocatalysts, where evolutionary adjustments in the structural color will tune the activity of the surface to achieve a selective catalytic degradation of different pollutants.

## 2. Materials and Methods

### 2.1. Butterflies

**Figure 1:**
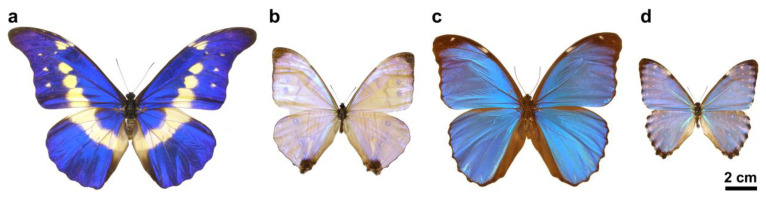
Photographs of male *Morpho* specimens in dorsal view. (**a**) *M. rhetenor helena*, (**b**) *M. sulkowskyi*, (**c**) *M. menelaus*, (**d**) *M. portis* are shown.

The butterfly samples were obtained from the curated Lepidoptera collection of the Institute of Technical Physics and Materials Science, Centre for Energy Research. Male specimens of four species representing the neotropical butterfly genus *Morpho* (Lepidoptera: Nymphalidae: Morphinae) were investigated [31,32]: *M. menelaus menelaus*, *M. rhetenor helena*, *M. portis thamyris*, *M. sulkowskyi calderoni* (Figure 1). None of the species used in this study were subjected to any restrictions. The flattest and most homogeneous 15 × 15 mm square samples were cut from butterfly wings and fixed on 20 × 20 mm glass substrates using heated polylactic acid (PLA) adhesive and 20 × 20 mm polytetrafluoroethylene (PTFE) frames with a 15 × 15 mm opening, similarly as the schematic image of Figure 1 in [22].

### 2.2. Atomic Layer Deposition (ALD)

Atomic layer deposition of 10, 15, and 20 nm thick ZnO layers onto the butterfly wings was carried out as it was described in [21]. A Picosun Sunale R-100 ALD reactor was used for the deposition with diethylzinc (DEZ) precursor and water vapor as oxidant. The carrier gas and purging medium was 99.999% purity nitrogen. Flow rates of the precursor gas and water vapor were 150 sccm. The pressure in the chamber was kept at 14 mbar during deposition. An ALD cycle for depositing ZnO layers consists of a 0.5 s pulse of DEZ and 15 s nitrogen purge, then followed by a 0.5 s pulse of water and 20 s nitrogen purge. As butterfly wings were thermally sensitive, the growth temperature was maintained at 100 °C. The growth rate at this temperature was 0.18 nm/cycle, therefore the 10 nm layer thickness required 60 pulses, the 15 nm layer 90 pulses, and the 20 nm layer 120 pulses.

The glass substrates (used as reference surfaces) were prepared by cutting them into the same size (15 × 15 mm) as the butterfly wing samples and were cleaned with acetone, isopropyl alcohol, and deionized water, respectively. The same ZnO coating process was applied as in the case of the butterfly wings.

### 2.3. Scanning Electron Microscopy (SEM)

Cryogenically prepared butterfly wing samples were cut immersed in liquid nitrogen using a surgical prep blade. The samples were fixed onto metal sample holders with conductive carbon tape and were examined via scanning electron microscopy using a Thermo Fisher Scientific Scios 2 DualBeam (Waltham, MA, USA) system.

### 2.4. X-ray Diffraction Analysis (XRD)

XRD measurements were carried out with a D8 Discover X-ray diffractometer (Bruker AXS, Karlsruhe, Germany) equipped with Göbel-mirror and scintillation detector using Cu Kα (λ = 1.5406 Å) radiation. The X-ray beam dimensions were 1 mm × 5 mm, and the 2Θ step size was 0.05°, measuring time 100 s/step.

### 2.5. Reflectance Spectrophotometry

Reflectance spectrophotometry measurements were conducted using a fiber optic Avantes AvaSpec-HSC1024 × 58TEC-EVO (Apeldoorn, The Netherlands) modular spectrophotometer. An Avantes AvaLight-DH-S-BAL stabilized UV-visible light source was used for illumination, and an Avantes AvaSphere-30-REFL integrating sphere for light collection. Reflectance measurements were carried out with respect to an Avantes WS-2 diffuse tile as a reference standard. The reflectance of butterfly wing samples was measured in the 200–950 nm wavelength range. Data analysis was performed using OriginPro 2021 (OriginLab Corporation, Northampton, MA, USA) software.

### 2.6. Photocatalytic Degradation Measurements

The photocatalytic activity was evaluated based on the removal of MO (50 µM) and RhB (15 µM) from an unbuffered solution upon illumination using Adrona ultrapure water as solvent. The initial dye concentrations were set by weighing in the commercial products of known purity (Reagent grade purity, Molar Chemicals Ltd., Halásztelek, Hungary). We made fresh stock solutions of both dyes that were kept in the dark (no detectable degradation, or pH change for ca. 1 week), and those were diluted to the required concentrations before each experiment. The pH of the solutions was measured as 5.7(1) for RhB and 4.9(1) for MO before and after the reactions by using an InLab Micro Pro-ISM pH sensor connected to a Mettler-Toledo InLab 731-ISM unit. The ZnO samples were placed vertically in 20 mL of the solution in a glass cuvette with magnetic stirring at room temperature. A heat-free 300 W Xe lamp (Asahi Spectra MAX-303, Torrance, CA, USA), with fiber optics, was applied as a light source supplying an adjustable beam (15 × 15 mm square-shaped illumination). The distance between the lamp and the catalytic surface immersed in the cuvette was 6 cm (corresponding to ~100 mW/cm^2^ light power). The emission spectrum of the light source and the transmittance of the glass cuvette are found in our preliminary work, see Figure 1a and Figure S1 within [21]. The dye degradation was followed by an Agilent Cary 60 UV–visible spectrophotometer equipped with an immersion probe (*l* = 1 cm) that was placed inside the solution, out of the illumination area. Spectra were collected every fifth minute during the 2-h reaction time. Degradation of MO and RhB was followed at the wavelength of absorption maxima at 463 nm (calc. *ε*_MO_ = 26.4(4) × 10^3^ M^−1^ cm^−1^) (Figure S1) and 554 nm (calc. *ε*_RhB_ = 95.9(9) × 10^3^ M^−1^ cm^−1^) (Figure S2), respectively. The conversion data were calculated from each measured spectra as X = (A_0_ − A_i_)/A_0_, where A_0_ is the initial absorbance of the dyes at their peak wavelength, and A_i_ is the absorbance at *t* = i in the reaction. The reaction rates were obtained from the slope of the fitted linear to the X vs. *t* data points, as follows: rate (nmol/min) = c_dye_ (nmol/L) × V_r_ (L) × slope (1/min). The values are given in nmol/min in Table S1. Soaking tests of the surfaces in MO and RhB solution before their use ruled out any detectable role of initial adsorption of the dye in the change of absorbance (note the high reaction volume over catalytic surface area ratio).

### 2.7. Liquid Chromatography-Mass Spectroscopy (LC-MS)

The relevant intermediates from the degradation of MO and RhB were separated and identified using Agilent 1200 liquid chromatograph (LC) and Agilent 6410 quadrupole mass spectrometer (MS/MS) devices. The isocratic separation was implemented by a reverse phase C18 column (Phenomenex EVO C18 100 A, 2.6 µm, 100 × 3 mm, Torrance, CA, USA). The eluents were ammonium acetate and acetonitrile. The flow rate was 0.3 mL/min. The optimal MS parameters were as follows: gas temperature 350 °C, gas flow 12 L/min, nebulizer 25 psi, capillary voltage ±3500 V, and fragmentor voltage 140 V in positive ionization mode. In MS/MS fragmentation N_2_ was used as collision gas.

## 3. Results and Discussion

The males of the investigated *Morpho* species representing different hues of blue. *M. rhetenor helena* (dark blue), *M. sulkowskyi calderoni* (light blue), *M. menelaus menelaus* (dark greenish blue), and *M. portis thamyris* (light greenish blue) had iridescent structural colors on their dorsal wing surfaces originated from photonic nanoarchitectures characteristic of these species [31,32] (Figure 1).

**Figure 2:**
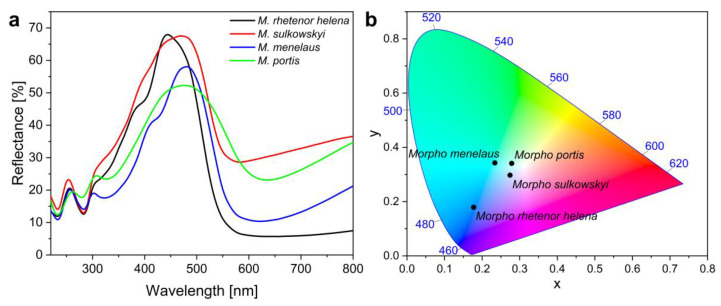
(**a**) Integrating sphere reflectance spectra of male *Morpho* specimens. (**b**) The measured spectra were converted into the CIE 1931 chromaticity diagram [34].

Integrating sphere reflectance spectrophotometry measurements were taken on the forewings of the specimens (Figure 2a) using a PTFE-based diffuse tile as a reference standard. The integrating sphere collects all light reflected the upper hemisphere from the sample through a spot 5 mm in diameter, which contains hundreds of cover scales, thus integrating the unique, highly angle-dependent reflectance. The measured wing reflectances cover a smaller wavelength range compared to males of genus *Arhopala* where a similar structural tuning of photonic nanoarchitectures was found [33], although the shape and the width of the reflectance peaks are significantly different from each other. This results in characteristically different hues of structural colors, which were found to be consistent with the photographs (Figure 1), when the measured reflectance spectra were converted into the CIE 1931 chromaticity diagram (Figure 2b) [34].

**Figure 3:**
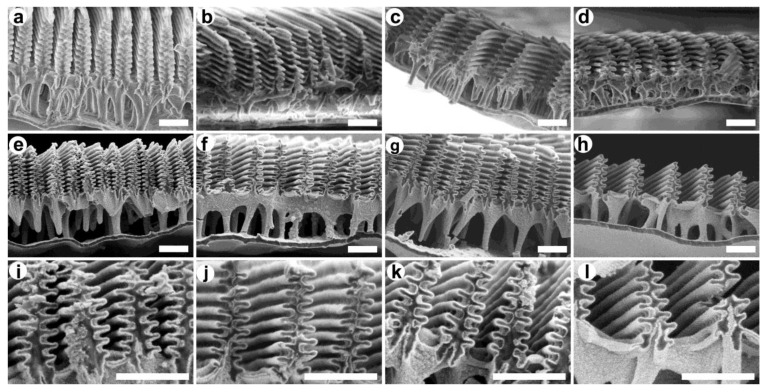
Cryogenically prepared cover scale sections of *Morpho* specimens in SEM. Images of the cover scales of the investigated species for (**a**,**e**,**i**) *M. rhetenor helena*; (**b**,**f**,**j**) *M. sulkowskyi*; (**c**,**g**,**k**) *M. menelaus*; and (**d**,**h**,**l**) *M. portis* are shown without (upper row) and with 20 nm ZnO coating (middle row) in the same magnification. Scale bar: 1 µm. High-magnification images of the ZnO-coated samples can be seen in the lower row. Scale bar: 1 µm.

Non-embedded samples were prepared cryogenically from the wings of each species and investigated using SEM. In Figure 3a–d, the images of the cover scale sections can be seen that contain the photonic nanoarchitectures characteristic of *Morpho* species [31,32]. Due to the lamellar structure of the ridges, these can be highly reflective and generate intensive structural colors (Figure 2), and they also have a large specific surface area. Therefore, it is worth using them as biotemplates, i.e., a substrate for semiconductor thin film coating.

**Figure 4:**
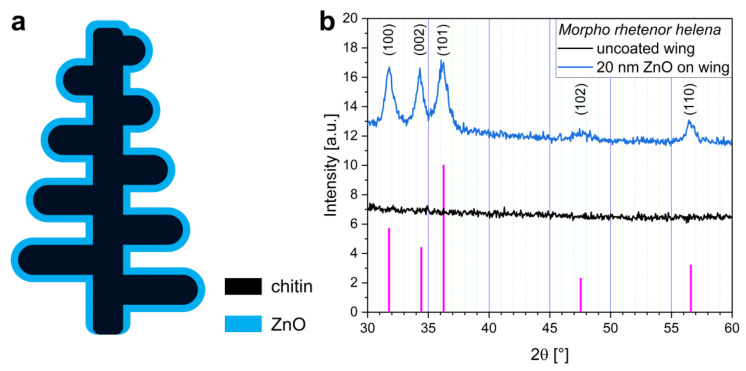
(**a**) Schematics of a *Morpho*-type nanostructure cross-section with the as-deposited ZnO thin film. (**b**) X-ray diffractogram shows a nanocrystalline, wurtzite-type structure of 20 nm ZnO deposited on the *Morpho rhetenor helena* wing-scale nanoarchitecture. Purple lines mark the XRD pattern of hexagonal, wurtzite-type ZnO. The uncoated wing was measured as a reference.

ZnO is one of the semiconductors that can be grown on the surface of the biological photonic nanoarchitectures using low-temperature atomic layer deposition. This way the micro- and nanoscale features of the chitinous cover scales are well preserved by the thin films without the need for a post-annealing process which would damage the biological material. XRD measurements showed that the as-deposited ZnO had a nanocrystalline, wurtzite-type structure (Figure 4b) [9] on the butterfly wings. Based on optical transmittance measurements on glass substrates, the electronic band gap of 3.3–3.35 eV was estimated from the approximately 380 nm absorption edge of ZnO thin films (Figure S3) [21].

Recent results showed that ZnO is a favorable coating for visible light-driven photocatalysis when *Morpho* butterflies are applied as biotemplates, since together they can effectively improve the degradation rate of water-soluble pollutants or dyes [21]. Based on these, we deposited 10–15–20 nm thick ZnO coatings onto the nanoarchitectures of the investigated specimens. In Figure 3e–h, SEM images of the nanostructures with 20 nm ZnO coating can be seen with magnified parts in Figure 3i–l, which show that the 20 nm thick coating formed precisely and homogeneously on the surface of the photonic nanoarchitectures as in the schematic drawing of Figure 4a. Magnified versions with thickness measurements of Figure 3i–l are shown in Figure S4.

**Figure 5:**
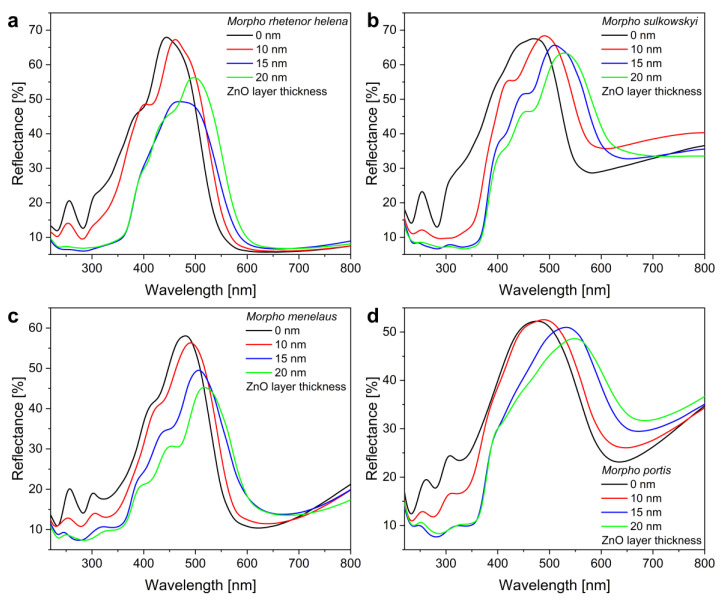
Reflectance spectra of the investigated *Morpho* specimens when 10–15–20 nm thickness of ZnO thin films were deposited: (**a**) *M. rhetenor helena*, (**b**) *M. sulkowskyi*, (**c**) *M. menelaus*, and (**d**) *M. portis* are shown.

The photonic nanoarchitectures of the different species have the same basic structure, therefore the deposited ZnO layers tuned their structural color in a similar manner. It is worth noting in the measured spectra (Figure 5) that the reflectance peaks were uniformly shifted to the infrared with the thickening of the ZnO coating. As ZnO thin films have a refractive index of 1.5–1.6 in the visible wavelength range [35], the deposition increased the thickness of the high refractive index component (chitin, n = 1.56 [36]) of the photonic nanoarchitecture, resulting in the redshift of the reflectance peaks. The reflectance spectra can be also used to examine the homogeneity of the deposited ZnO layers on hundreds of cover scales simultaneously, compared to SEM where only a very small sample volume can be investigated. By monitoring the absorption edge of the ZnO below 380 nm, the quality of the different deposited layers can be compared. Apparently, the 10 nm ZnO deposition did not form a conformal and homogeneous coating on the photonic nanoarchitectures, as significant intensity in the UV range of the reflectances persisted. However, when 15 nm and 20 nm coatings were applied, an almost perfect UV absorption of the biotemplated photonic nanoarchitectures can be seen.

**Figure 6:**
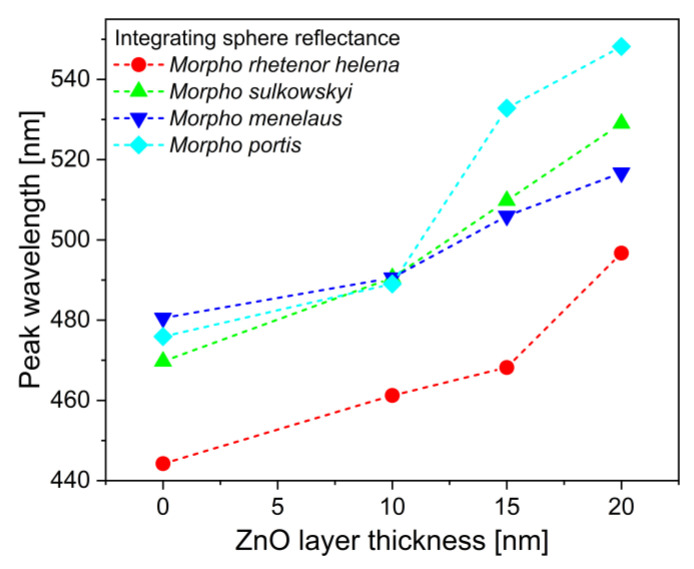
Peak shift of the reflectance spectra of the investigated *Morpho* species with the increasing thickness of the deposited ZnO layer. *M. rhetenor helena*, *M. sulkowskyi*, *M. menelaus*, and *M. portis* are shown in pristine states and when 10–15–20 nm of ZnO thin films were deposited.

The spectral shifts of the four species are compared in Figure 6, where the peak wavelengths were plotted in the function of the deposited ZnO layer thickness. One can see that the 10 nm thin films shifted the reflectance maxima less, suggesting that the deposition was not completely homogeneous in this case, while the 15–20 nm thick layers caused a more significant redshift for the same increment in the layer thickness. However, ZnO deposition cannot be increased to any extent without losing the photonic band gap: the bottom row in the SEM images (Figure 3i–l and Figure S4) shows that even more than 20 nm thick layers would be impractical as they could fill the space between the lamellae, both eliminating the photonic band gap of the samples and reducing the effective surfaces area of the nanoarchitectures. Finally, the structure of the *M. portis* species is visibly slightly different from the others, as it has fewer lamellae in the perforated multilayer. This was also reflected in the interaction with the deposited ZnO thin films: the structural color shift had a slightly different character compared to the other three species, which had smaller peak wavelength differences between the 10 nm and the 15 nm thick ZnO layers.

**Figure 7:**
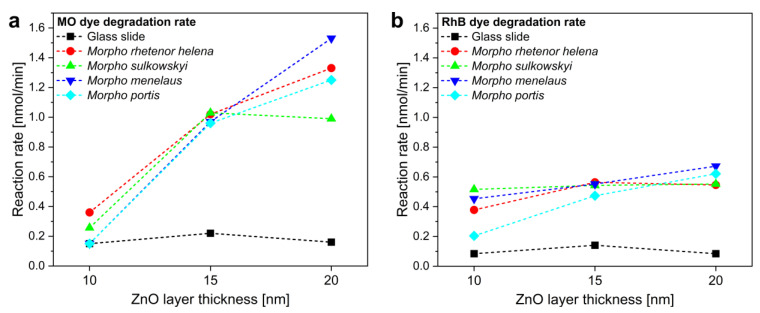
Reaction rate versus ZnO layer thickness for glass slide substrates and *Morpho* butterfly wings when (**a**) MO or (**b**) RhB test dyes were decomposed upon visible light illumination. *M. rhetenor helena*, *M. sulkowskyi*, *M. menelaus*, and *M. portis* are shown.

The photocatalytic activity of the *Morpho* butterfly wings with ZnO coatings was tested using the aqueous solutions of MO and RhB dyes in a glass cuvette as a test reactor upon visible light illumination. The absorbance of the solutions was monitored using an immersion probe in the first 2 h of the illumination, in which period the conversion of the dyes was low thus enabling linear fit to the time-dependent conversion data points [21]. The photodegradation rates were referenced to that of ZnO-coated glass slides with the same layer thickness (10–15–20 nm), while unmodified butterfly wings were also investigated as control samples. The uniform and homogeneous light intensity by the lamp and reactor setup and the planar geometry of the butterfly wings and the reference glass substrates together allowed the direct comparison of the photocatalytic activity. The reaction rate was measured for each sample and the results were summarized in Figure 7 and Table S1.

The reference glass substrates with 10–15–20 nm ZnO thin films showed low photocatalytic activity (reaction rate <0.2 nmol/min) compared to the butterfly wing-based samples. The unmodified butterfly wings had reaction rates of 0.240 and 0.222 nmol/min for MO and RhB, respectively, which may be the result of the increased specific surface area. When *Morpho* wings with 10 nm ZnO thin film coatings were investigated, moderate increments of photocatalytic activity were found compared to the pristine wings. This may be a direct consequence of the inhomogeneity and nonconformal deposition of the 10 nm ZnO thin film. When the other layer thicknesses were investigated, an increased degradation rate was observed for both test dyes. This enhancement was more prominent in the case of MO dye compared to RhB: we measured almost a ten-fold increase of MO degradation rate when the 20 nm ZnO coated *M. menelaus* wings were compared to a glass slide with the same ZnO coating, while with RhB, the maximal enhancement was only eight-fold for the same samples. For a deeper understanding of the optical interaction between the photonic band gap of the biotemplated samples and the absorption band of the test dyes, the reaction rates were investigated separately for each butterfly species as a function of the peak wavelength of the measured structural color.

**Figure 8:**
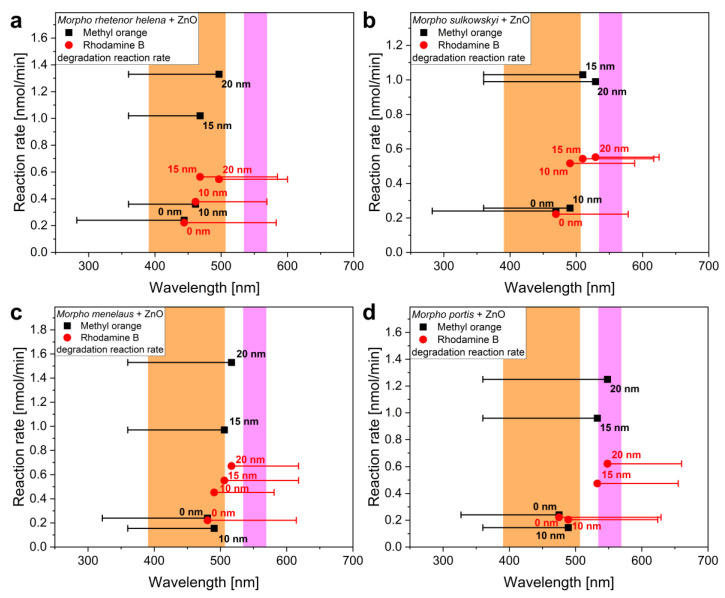
Reaction rates plotted against the peak wavelength of the wing reflectance for each species: (**a**) *M. rhetenor helena*, (**b**) *M. sulkowskyi*, (**c**) *M. menelaus*, (**d**) *M. portis*. The bars show the width of the red/blue edges of the reflectance peaks measured on the corresponding spectra of Figure 5, while the labels show the as-deposited ZnO thin film thickness used in the given measurement. The areas in orange correspond to the absorption band of MO, while the areas in magenta are that of RhB.

In Figure 8, the reaction rates were plotted against the peak wavelength of the wing reflectance for each species and for each ZnO thickness. The orange and magenta areas in the plot show the absorption bands of MO and RhB, respectively. The horizontal bars of the peak wavelength data points represent the width of the blue and the red edges of the corresponding reflectance spectra (Figure 5). Both blue and red edge wavelength widths were measured between the peak wavelength and wavelength of the blue/red boundaries of the reflectance peaks, respectively. In the case of the two test dyes, only the width of the relevant edge is shown for easier understanding: in the case of MO, the blue edge of the structural color, while in the case of RhB, the red edge is shown. As seen in all four cases, if the blue or red edge of the photonic band gap coincides with the edge of the absorption band of the dye, the catalytic efficiency considerably increases. However, when the reflectance maximum—and its close surroundings—of the photonic nanoarchitecture falls within the region of dye absorption maximum, then we experience a gradual decrease in the reaction rate. The enhancement of the catalytic efficiency is attributed to the slow light effect, which is a characteristic light-matter interaction in photonic band gap materials where incident photons are significantly slowed at the blue and red boundaries of the photonic band gap wavelength ranges, thus facilitating the absorption of incident photons by the semiconductor [29,37]. Nevertheless, the performance of the photocatalysts decreases when the photonic stop band is shifted within the absorbance band of the dyes due to increased light screening by the dye molecules, which greatly reduces the intensity of light falling onto the biotemplated semiconductor nanoarchitecture.

The interaction of the photonic band gap and the absorption of the test dyes is worth investigating separately for each dye. In the case of MO, none of the samples with 10 nm ZnO layers showed improvement compared to the pristine wings. This may be related to the inhomogeneous or nonconformal deposition of ZnO thin films, which resulted in poor photocatalytic performance. However, at 15 and 20 nm layer thicknesses, as the reflectance maximum was shifted away from the absorption band of the dye, the catalytic efficiency increased significantly as more and more slow photons were utilized in the excitation of the semiconductor. Here, there was no large-scale color change that would cause the loss of catalytic activity by the separation of the photonic band gap and the absorption band of MO. In the case of RhB, the tuning of the color of the butterflies by ZnO thin films shifted the photonic band gap into the absorption band of the dye, which is just the opposite of what happened in the case of MO. As a result, the growth in catalytic efficiency was far from what we have experienced in the case of MO; in the case of 15–20 nm thin films, an increase with a slowing tendency was often visible, since the absorption of the dye molecules reduced the number of slow photons capable of generating excitations in ZnO via screening.

In summary, the redshifting structural colors of the *Morpho* wings due to the deposited ZnO thin films resulted in opposite effects in the case of the two types of test dyes. When MO was degraded, the photocatalytic efficiency was enhanced with the increasing layer thicknesses due to the structural color shifting away from the absorption band of the dye, while in the case of RhB, the redshift caused increasing overlap of the peak reflectance and the dye absorption band which resulted in the saturation of the degradation rate enhancement.

**Figure 9:**
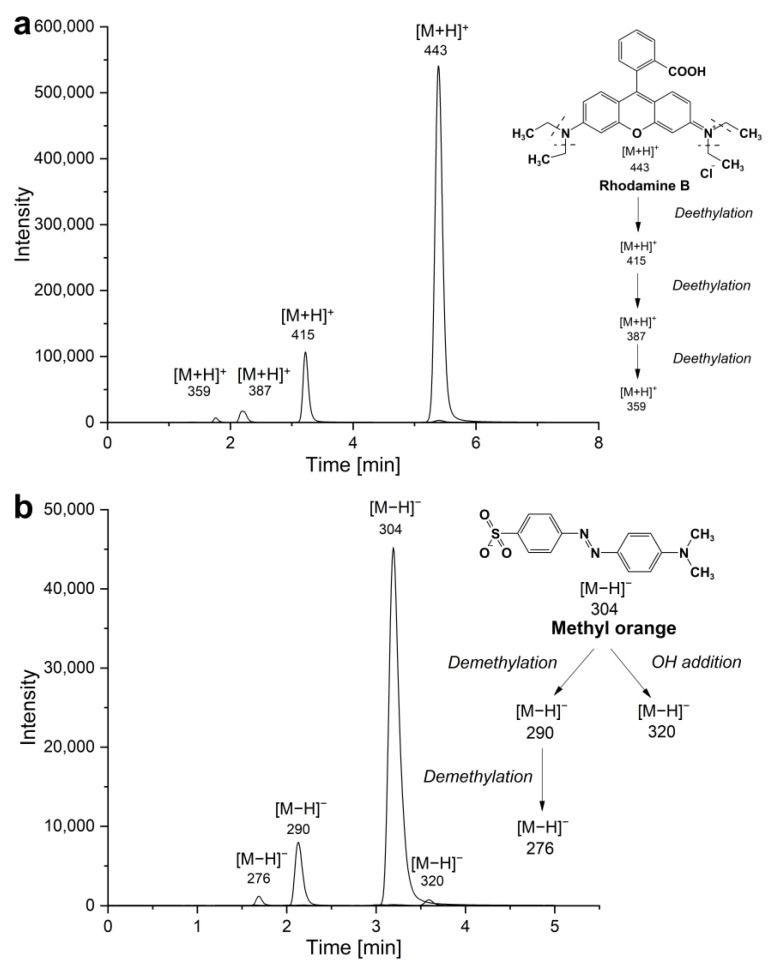
Selected ion chromatograms of products in (**a**) 15 µM RhB and (**b**) 50 µM MO solutions. Insets: Suggested reaction scheme based on identified products in degradation of (**a**) RhB and (**b**) MO, respectively.

Based on LC-MS analysis, the degradation products of RhB can be identified, and the degradation pathway proposed (Figure 9a). The RhB with *m/z* of 443 (t_r_ = 5.39 min) undergoes gradual *N*-de-ethylation, until losing its four *N*-alkyl groups in accordance with literature [38,39]. From mass spectra analysis, the intermediates were identified as *N*,*N*-diethylrhodamine (*m/z* = 415, t_r_ = 3.22 min), *N*-ethyl-*N′*-ethylrhodamine (*m/z* = 387, t_r_ = 2.21 min) and *N*-ethylrhodamine (*m/z* = 359, t_r_ = 1.76 min). The parent molecule of MO gives a signal at *m/z* of 304 (t_r_ = 3.21 min). In the degradation of MO (Figure 9b), there are two decomposition routes based on the identified intermediates: demethylation and hydroxyl addition on the aromatic ring [40]. The bonding energy for the –C–N bond of the amine group and –N=N– are 305 kJ/mol and 418 kJ/mol, respectively, thus it is not surprising that the process of demethylation is the preferred reaction at the early stage of the degradation [41]. The peaks of *m/z* 290 (t_r_ = 2.15 min) and *m/z* 276 (t_r_ = 1.71 min) correspond to the products of the successive demethylation. In the process of OH addition, the intermediate with *m/z* of 320 (t_r_ = 2.15 min) can be detected. The products identified may undergo further degradation via the scission of the diazenyl chromophore group. The above direct observations on the degradation products and the lack of hydroxylated products are in line with the conclusions from our earlier experiments using hydroquinone (HQ) as a substrate that excluded the involvement of photogenerated oxygen radicals and rather suggested dye sensitization during photodegradation [21].

## 4. Conclusions

Four *Morpho* species with similar photonic nanoarchitectures in the dorsal cover scales but with different hues of structural colors were used as biotemplates for ZnO thin film deposition. Using MO and RhB as test dyes, we investigated the effect of optical and structural tuning of the biotemplated butterfly wings conformally coated with ZnO on the photocatalytic performance upon visible light illumination. When MO was used, a considerable enhancement in the reaction rate was observed: we measured almost a ten-fold increase of the photocatalytic activity, 1.53 nmol/min when the 20 nm ZnO-coated *M. menelaus* wing was compared to the performance of the similarly coated glass slide (0.16 nmol/min). Using RhB, a saturating relationship was found between the degradation rate and the thickness of the deposited ZnO on butterfly wings. The shift in reactivity was associated with the spectral overlap between the ZnO-coated *Morpho* butterfly wing reflectance and the absorption band of the dyes. The enhancement in catalytic efficiency could be attributed to the slow light effect, where incident photons were significantly slowed at the blue and red boundaries of the photonic band gap wavelength ranges, thus facilitating light-induced changes in the surface-adsorbed dye molecules. However, in the case of RhB, the performance enhancement of the photocatalysts decreased when the photonic band gap was shifted within the absorbance band of the dye, due to increased light screening of the dye molecules, which in turn greatly reduced the intensity of light that could interact with the biotemplated semiconductor nanoarchitectures. This way, biotemplated ZnO photonic nanoarchitectures can be fabricated in which the structural color can be tuned to the absorption band edge of the molecules to be degraded, thus enhancing photocatalytic efficiency.

## Data Availability

All relevant data analyzed during this study are included in this published article and its Supplementary information files. Raw datasets used during the current study are available from the corresponding author on reasonable request.

## Supplementary Materials

The following supporting information can be downloaded at: https://www.mdpi.com/article/10.3390/ma16093584/s1, Table S1: Reaction rates versus ZnO layer thickness for glass substrates and *Morpho* butterfly wings when MO or RhB test dyes were decomposed upon visible light illumination. Figure S1: Typical UV-vis absorption spectra of the MO solution recorded during photodegradation using a *Morpho menelaus* wing coated with conformal ZnO in 20 nm thickness (c_0_ = 50 μM). The arrows show the direction of spectral changes. Figure S2: Typical UV-vis absorption spectra of the RhB solution recorded during photodegradation using a *Morpho menelaus* wing coated with conformal ZnO in 20 nm thickness (c_0_ = 15 μM). The arrow shows the direction of spectral changes. Figure S3: The electronic band gap of ZnO was estimated by the transmittance measurements of deposited ZnO thin films on glass substrates. From the transmittance measurements (top left), the normalized transmittances of the deposited ZnO layers (top right) can be calculated, to which only the absorbance of the ZnO thin films contributes. Tauc plots of 15 nm and 20 nm ZnO thin films deposited to glass substrate can be see below. Based on the linear fits (see dotted lines), electronic band gaps of 3.3–3.35 eV can be estimated for the deposited ZnO layers, which is in good agreement with the literature values [42]. Figure S4: Cryogenically prepared cover scale sections of *Morpho* specimens in SEM. Images of the cover scales of the investigated species for (a) *M. rhetenor helena*; (b) *M. sulkowskyi*; (c) *M. menelaus*; and (d) *M. portis* are shown with 20 nm ZnO coating. Scale bar: 500 nm.
