# Supplementary material for: Investigating the Effect of Reflectance Tuning on Photocatalytic Dye Degradation with Biotemplated ZnO Photonic Nanoarchitectures Based on Morpho Butterfly Wings
Source: Materials (Basel). 2023 May 7;16(9):3584. doi: 10.3390/ma16093584 (PMC10179795; doi:10.3390/ma16093584)
Supplement: Supplementary file 1 [file materials-16-03584-s001.zip › materials-2325370-supplementary.pdf]

Supplementary Information

Investigating the Effect of Reflectance Tuning on Photocatalytic Dye Degradation with Biotemplated ZnO Photonic Nanoarchitectures Based on *Morpho* Butterfly Wings

Gábor Piszter, Gergely Nagy, Krisztián Kertész, Zsófia Baji, Krisztina Kovács, Zsolt Bálint, Zsolt Endre Horváth, József Sándor Pap, László Péter Biró

Table S1. Reaction rates versus ZnO layer thickness for glass substrates and *Morpho* butterfly wings when MO or RhB test dyes were decomposed upon visible light illumination.

		**Reaction rate** [nmol/min]				
	**ZnO layer thickness** [nm]	**Glass**	***Morpho portis***	***Morpho rhetenor helena***	***Morpho menelaus***	***Morpho sulkowskyi***
**Methyl orange**	10	0.15(3)	0.15(1)	0.36(2)	0.15(1)	0.257(20)
	15	0.22(5)	0.96(2)	1.02(1)	0.97(1)	1.03(2)
	20	0.16(3)	1.25(1)	1.33(1)	1.53(1)	0.99(1)
**Rhodamine B**	10	0.084(5)	0.204(3)	0.378(5)	0.453(9)	0.516(15)
	15	0.141(15)	0.474(8)	0.564(15)	0.552(10)	0.543(8)
	20	0.084(6)	0.621(9)	0.546(8)	0.672(7)	0.552(13)

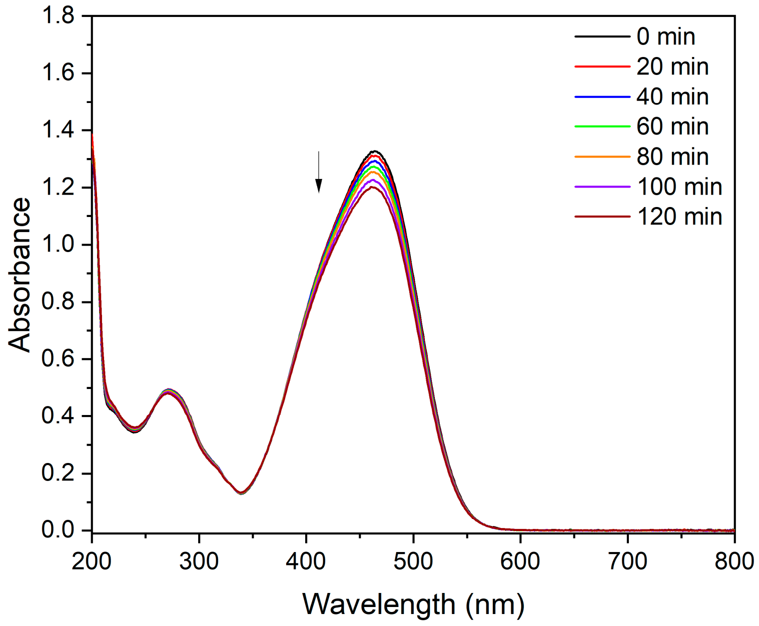

Figure S1. Typical UV-vis absorption spectra of the MO solution recorded during photodegradation using a *Morpho menelaus* wing coated with conformal ZnO in 20 nm thickness ($c_0 = 50\ \mu M$). The arrows show the direction of spectral changes.

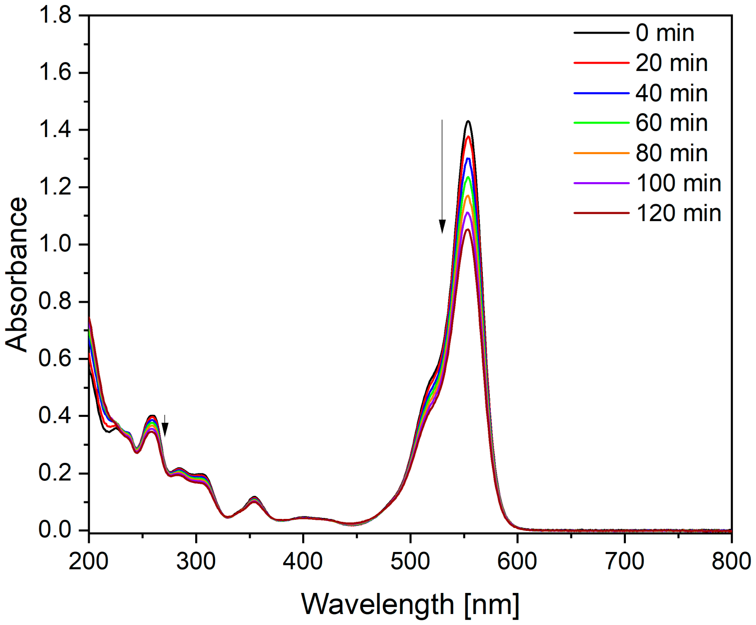

Figure S2. Typical UV-vis absorption spectra of the RhB solution recorded during photodegradation using a *Morpho menelaus* wing coated with conformal ZnO in 20 nm thickness ($c_0 = 15\ \mu M$). The arrow shows the direction of spectral changes.

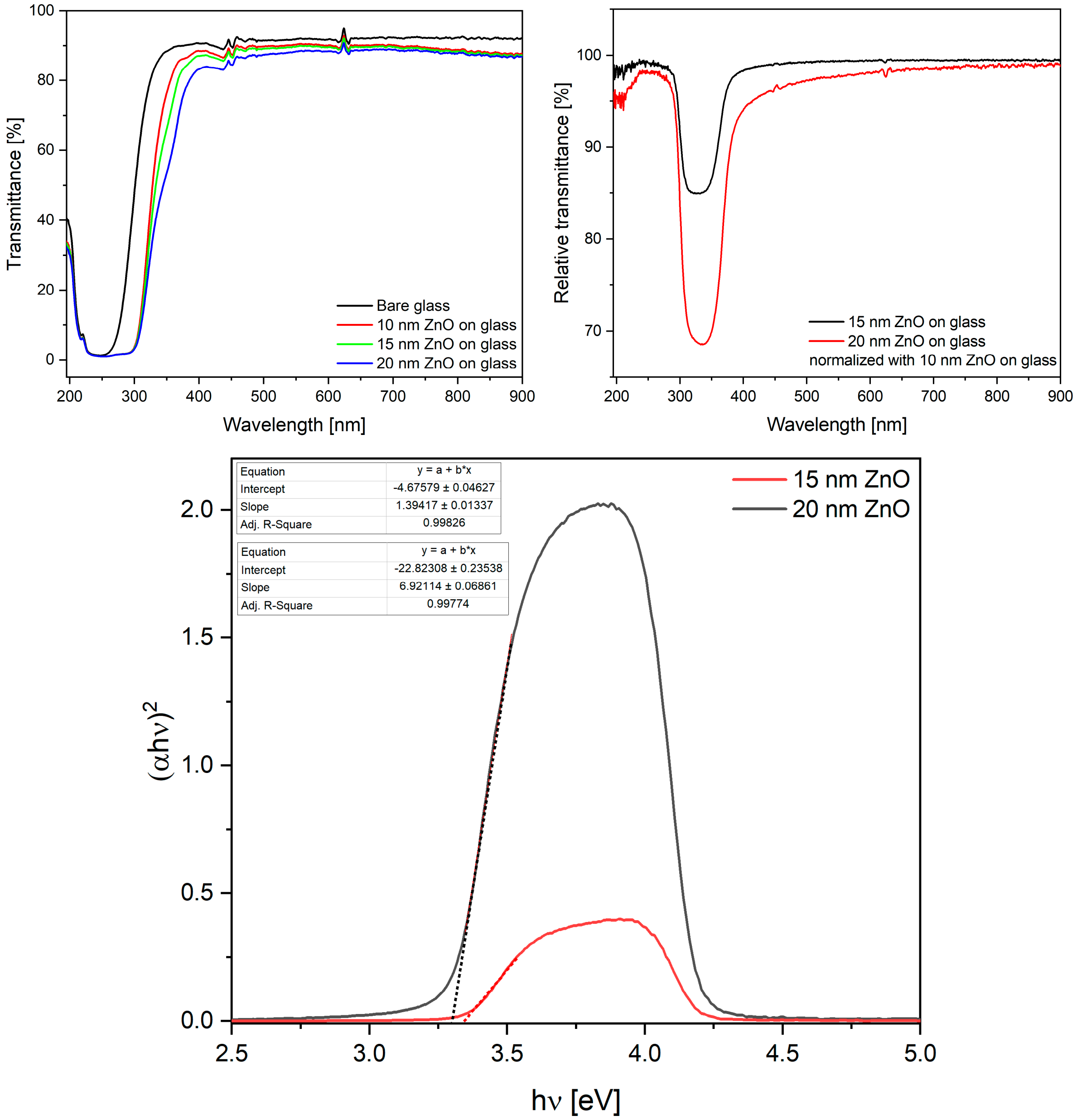

Figure S3. The electronic band gap of ZnO was estimated by the transmittance measurements of deposited ZnO thin films on glass substrates. From the transmittance measurements (top left), the normalized transmittances of the deposited ZnO layers (top right) can be calculated, to which only the absorbance of the ZnO thin films contributes. Tauc plots of 15 nm and 20 nm ZnO thin films deposited to glass substrate can be see below. Based on the linear fits (see dotted lines), electronic band gaps of 3.3–3.35 eV can be estimated for the deposited ZnO layers, which is in good agreement with the literature values [1].

References:

[1] Janotti, A.; Van de Walle, C.G. Fundamentals of zinc oxide as a semiconductor. *Rep. Prog. Phys.* **2009**, *72*, 126501. DOI:10.1088/0034-4885/72/12/126501

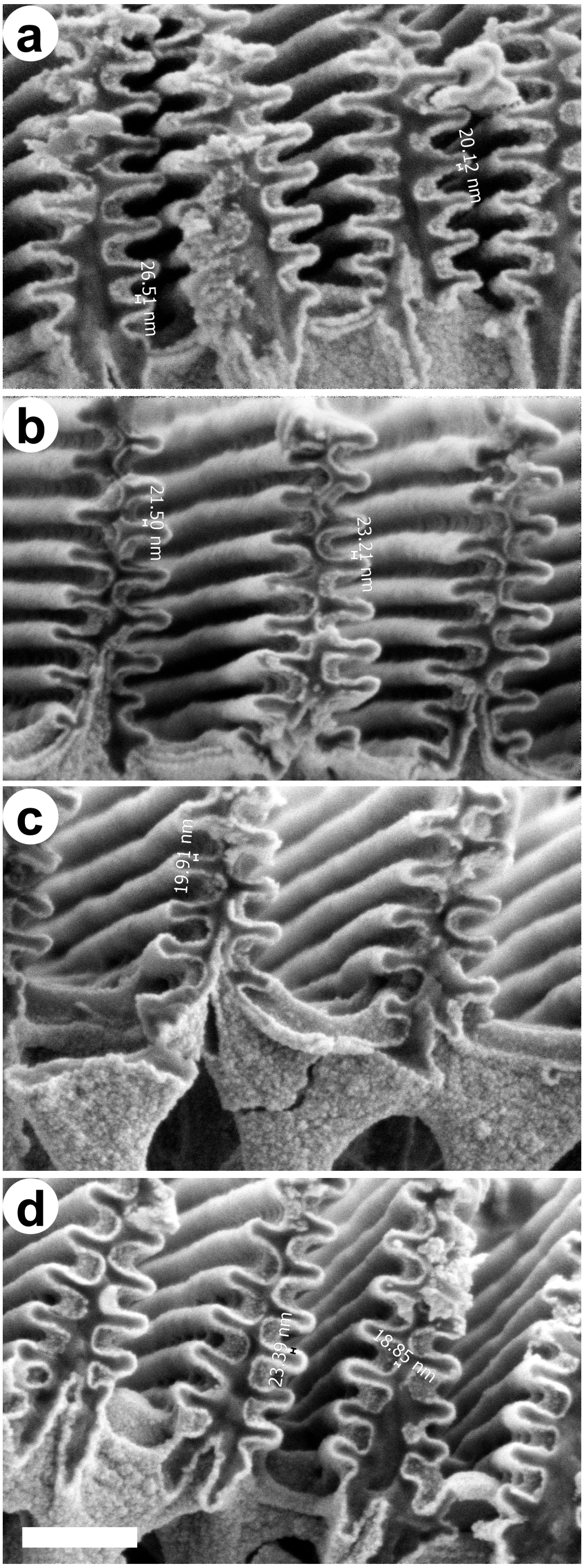

Figure S4. Cryogenically prepared cover scale sections of *Morpho* specimens in SEM. Images of the cover scales of the investigated species for (**a**) *M. rhetenor helena*; (**b**) *M. sulkowskyi*; (**c**) *M. menelaus*; and (**d**) *M. portis* are shown with 20 nm ZnO coating. Scale bar: 500 nm.
